# A Functional and Immunologic Point of View on Corneal Endothelial Transplantation: A Systematic Review and Meta-Analysis

**DOI:** 10.3390/jcm13123431

**Published:** 2024-06-12

**Authors:** Sara Spelta, Alessandra Micera, Daniele Gaudenzi, Matteo Niutta, Pier Luigi Surico, Antonio De Vincentis, Marco Coassin, Antonio Di Zazzo

**Affiliations:** 1 Department of Ophthalmology, University Campus Bio-Medico, 00128 Rome, Italy; s.spelta@unicampus.it (S.S.); danielegaudenzi93@gmail.com (D.G.); matteoniutta@libero.it (M.N.); pierluigi.surico@gmail.com (P.L.S.); m.coassin@policlinicocampus.it (M.C.); 2 Fondazione Policlinico Campus Bio-Medico, 00128 Rome, Italy; 3 Research and Development Laboratory for Biochemical, Molecular and Cellular Applications in Ophthalmological Sciences, IRCCS–Fondazione Bietti, 00184 Rome, Italy; alessandra.micera@fondazionebietti.it; 4 Internal Medicine, University Campus Bio-Medico, 00128 Rome, Italy

**Keywords:** endothelial transplantation, cornea, DMEK, DSAEK, UT-DSAEK

## Abstract

**Background:** To systematically review and meta-analyze the immunologic aspects and outcomes of various endothelial keratoplasty (EK) techniques, specifically comparing Descemet’s Stripping Automated Endothelial Keratoplasty (DSAEK), Ultra-Thin Descemet’s Stripping Automated Endothelial Keratoplasty (UT-DSAEK), and Descemet’s Membrane Endothelial Keratoplasty (DMEK). **Methods:** Systematic review and meta-analysis. Main outcomes were the proportion of patients achieving a best spectacle-corrected visual acuity (BSCVA) of 20/20 at 6 months after keratoplasty, rejection rate one year after surgery, BSCVA at last follow up, and postoperative immunomodulating regimen. **Results:** A higher proportion of DMEK patients achieved a BSCVA of 20/20 after 6 months. UT-DSAEK and DMEK showed similar rejection rates with a lower risk of re-bubbling for UT-DSAEK (4% vs. 20%). **Conclusions:** DMEK showed faster visual recovery than UT-DSAEK but a similar rejection rate and long-term visual acuity. One-year postoperative slow tapering steroid regimen has a positive but not (yet) significant effect on rejection risk and visual outcomes.

## 1. Introduction

Corneal transplantation is the most successful tissue transplantation procedure performed in humans [1,2].

Since the first penetrating keratoplasties (PK) transplantation, techniques have been continuously improved to the level of lamellar surgeries in order to reduce the immunogenicity of transplanted tissue, hence the risk of rejection, and ultimately to increase corneal graft survival [3].

In the cornea and anterior chamber, several active mechanisms of immune tolerance contribute to a high success rate of transplantation [4,5,6]. Anterior chamber-associated immune deviation (ACAID) acts in preventing the development of delayed-type hypersensitivity responses to non-self antigens in the anterior chamber [7,8,9,10].

This process is particularly active and effective in corneal grafts performed in non-inflamed, non-vascularized host beds, which are devoid of lymphatic vessels, thus classified as low-risk transplants (LR) [11]. However, despite the high success rate of low-risk LR corneal transplantation, 18–30% of transplanted corneas experience at least one episode of immune rejection. Of these, 2.3% to 68% may worsen into severe immune rejection, compromising the endothelium and leading to eventual graft failure in one-third of these cases [11,12,13].

While PK boasts a generally high success rate, it remains an invasive procedure fraught with risks such as heightened rejection rates and anatomical complications like eroding sutures, infections, irregular astigmatism, and graft dehiscence [14]. In contrast, EK presents a less invasive, closed-eye alternative, requiring a smaller incision, thus reducing the likelihood of complications such as wound dehiscence, induced astigmatism, suprachoroidal hemorrhage, synechiae, and infection [15]. Furthermore, EK preserves corneal innervation and sensation to a larger extent, ensuring better ocular surface integrity and significantly lower rates of immunologic rejection compared to PK [14,15].

While the numerous advantages of EK over PK are evident, EK procedures (particularly DMEK) present their own challenges. They are more technically demanding, and the intraoperative maneuver for placing the donor tissue in the correct orientation can be difficult. It has been suggested that 3D visualization systems or intraoperative optical coherence tomography could be useful adjunct tools [16,17,18].

Despite these challenges, EK has surpassed PK in popularity. Due to the high prevalence of endothelial dystrophies prompting transplantation, 55% of keratoplasties in the United States in 2019 addressed endothelial cell failure. Consequently, EK, performed in 89% of those cases, has been the predominant form of keratoplasty in the United States since 2012.

Therefore, in LR cases, surgical procedures may be a crucial step for preventing the risk of immune reaction, eventual rejection, and subsequent failure by reducing the amount of immunogenic-transplanted tissue [3,12,19]. Hence, EK has been evolving from Descemet’s stripping endothelial keratoplasties (DSEK), in which a graft was fairly irregular and with a highly immunogenic amount of stroma, to DMEK, in which a stroma-denuded, flat, and regular graft is harvested [20].

However, the performance, immunogenicity, and reproducibility of the different EK techniques are still controversial, leading to a lack of guidelines regarding their alternative clinical applications and indications [21,22].

Our aim is to investigate the clinical outcomes and rejection risk related to post-operative prophylaxis in EK alternative procedures performed in corneal immunological sanctuary.

## 2. Material and Methods

### 2.1. Selection Criteria and Search Methods

Medline (PubMed), Cochrane Library, Web of Science, Google Scholar, Scopus, and Embase online libraries were used and a selection of the available published studies on DSAEK, UT-DSAEK, and DMEK as EK alternative procedures from 1980 to 2021 was made. An analysis of the literature and writing of the manuscript was performed following Preferred Reporting Items for Systematic Reviews and Meta-Analyses (PRISMA) guidelines (http://www.prisma-statement.org/) (Supplementary Figure S1, PRISMA checklist). All published peer-reviewed randomized clinical trials, case series, and case reports, divided according to evidence level, were selected. There were no restrictions in language or publication status, although selection was limited to human study participants. Articles were not restricted to a special postoperative variable.

### 2.2. Study Selection

Outcome measures were the percentage of patients reaching 20/20 best spectacles corrected visual acuity (BSCVA) within six months after surgery, the patients’ BSCVA at last follow up, and the percentage of graft which experienced rejection, in view of the postoperative regimen. Not all studies evaluated all variables; at least 2 outcome parameters should have been studied to be eligible.

### 2.3. Data Synthesis and Analysis

Meta-analysis was performed to investigate the occurrence of selected outcomes in patients undergoing DSAEK, UT-DSAEK, or DMEK either in comparison each other (when data are available) or in non-comparative studies. Odds ratios (ORs) and raw, i.e., untransformed, proportions were used to report the pooled effect of each EK technique on the outcome probability in comparative and non-comparative studies, respectively. Heterogeneity was evaluated using the Q statistic, expressed as the *p* value for the c2 test under the null hypothesis that the between-study variance (t2) equals 0, and the I2 test. Accordingly, random effect models were applied in the presence of significant heterogeneity (defined as I2 > 55% and/or a Q statistic *p* value below 0.05). Fixed effect models were used in the absence of heterogeneity. Subgroup analyses and meta-regressions were performed to evaluate the impact of selected moderators on the pooled effect sizes. Finally, the likelihood of methodological bias among included studies was estimated with a visual inspection of the funnel plot. Analyses were conducted using metafor and meta packages in R 4.0.2 (R Foundation for Statistical Computing, Vienna, Austria).

## 3. Results

A total of 211 manuscripts were screened, while 163 were evaluated, since only these focused on EK management. Only 25 clinical studies, particularly 4 randomized clinical trials, 1 open prospective clinical trial, and 20 retrospective studies, were included in our revision (Table 1).

Study data from 25 studies about EK alternative procedures were finally evaluated and reported in the analysis. The overall results are summarized in Table 2.

The main indications for EK were Fuchs endothelial corneal dystrophy (FECD), pseudophakic/aphakic bullous keratopathy, and secondary graft failure [19]. Rarely, EK is performed for other forms of endothelial dystrophy, such as posterior polymorphic corneal dystrophy (PPCD) or congenital hereditary endothelial dystrophy (CHED), for irido-corneo-endothelial syndrome (ICE), and for central Haab striae-related edema in buphthalmos [20].

**Figure 1:**
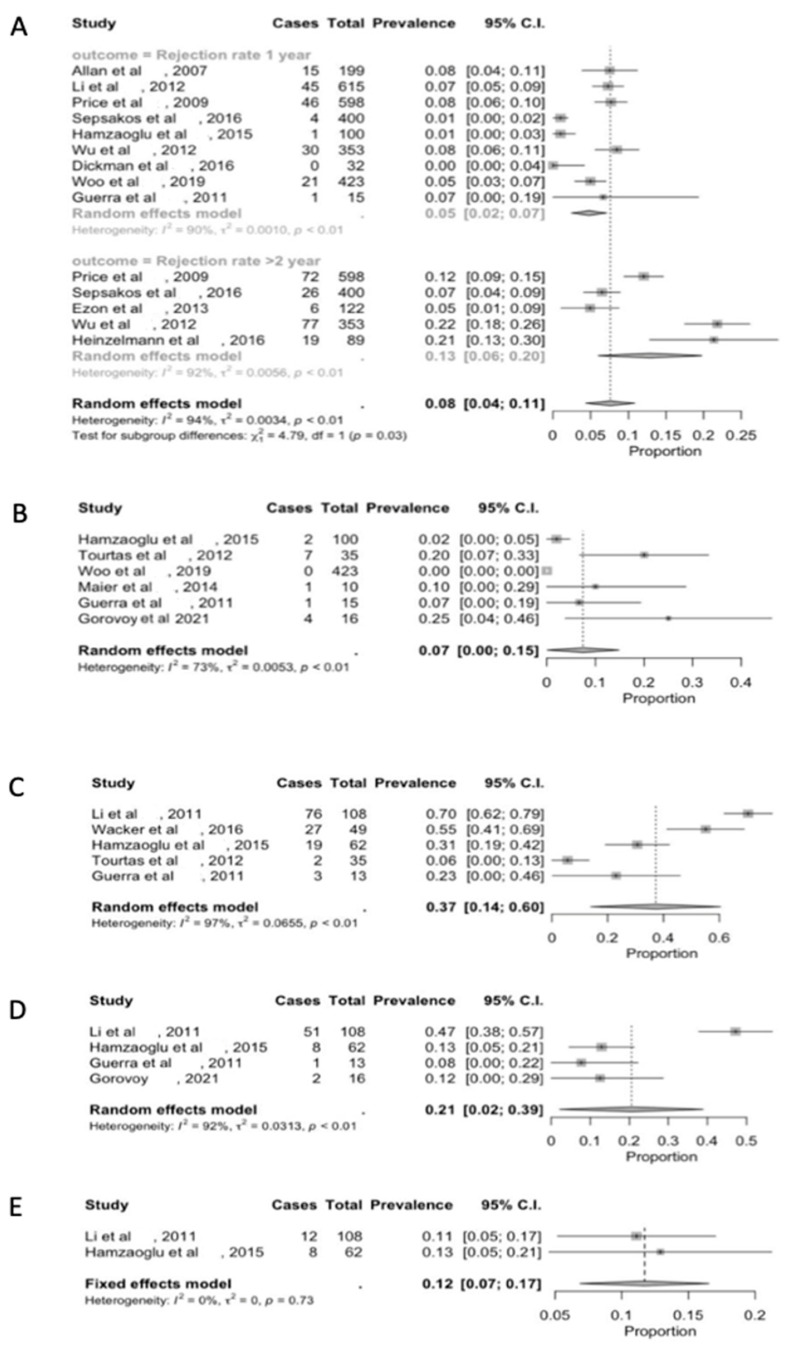
Forest plot diagram of DSAEK clinical outcomes: (**A**) rejection rate; (**B**) re-bubbling rate; (**C**) BSCVA > 20/30; (**D**) BSCVA 20/20; (**E**) success rate.

The DSAEK technique leads to a risk of 5% (C.I. 2–7) immune rejection at 1 year, which increases to 13% (C.I. 6–20) after 2 years (Figure 1A). These endothelial immune responses are clinically subtle and asymptomatic, although corneal edema and anterior chamber cells occur in 10–25% of cases [21,22,23,24,32,34,45]; endothelial rejection Khodadoust lines are rare. Generally, isolated precipitates, focal or diffuse, have been assessed (60–70%) [23,24,29,46,47,48].

However, graft rejection risk seems higher in eyes with pre-existing glaucoma and steroid responders and in African Americans [23]. The incomplete adhesion of the transplanted DSAEK graft is quite common. Such a complication requires a re-bubbling procedure [33,49], but at a much lower rate compared to other endothelial keratoplasty techniques, specifically around 7% (C.I. 0–15) of surgeries, even by novice surgeons (Figure 1B). A mean post-DSAEK hyperopic shift of 1.13 dioptres has been calculated [49,50]. This represents a faster visual recovery compared to PK, with 12% (C.I. 7–17) of patients achieving a BSCVA of 20/20 at six months after surgery. Instead, a BSCVA of 20/30 has been achieved in 37% (C.I. 14–60) of patients [25,26], while in 21% (C.I. 2–39), 20/20 is achieved at the last follow up (Figure 1C–E).

Postoperative regimen is highly variable among surgeons. However, dexamethasone phosphate 0.1% and prednisolone acetate 1% are commonly advised with a mean prophylaxis period of 1 year [27,28,49]. Medication type, therapy duration, or tapering schedule do not affect rejection risk and visual outcomes in the DSAEK procedure.

**Figure 2:**
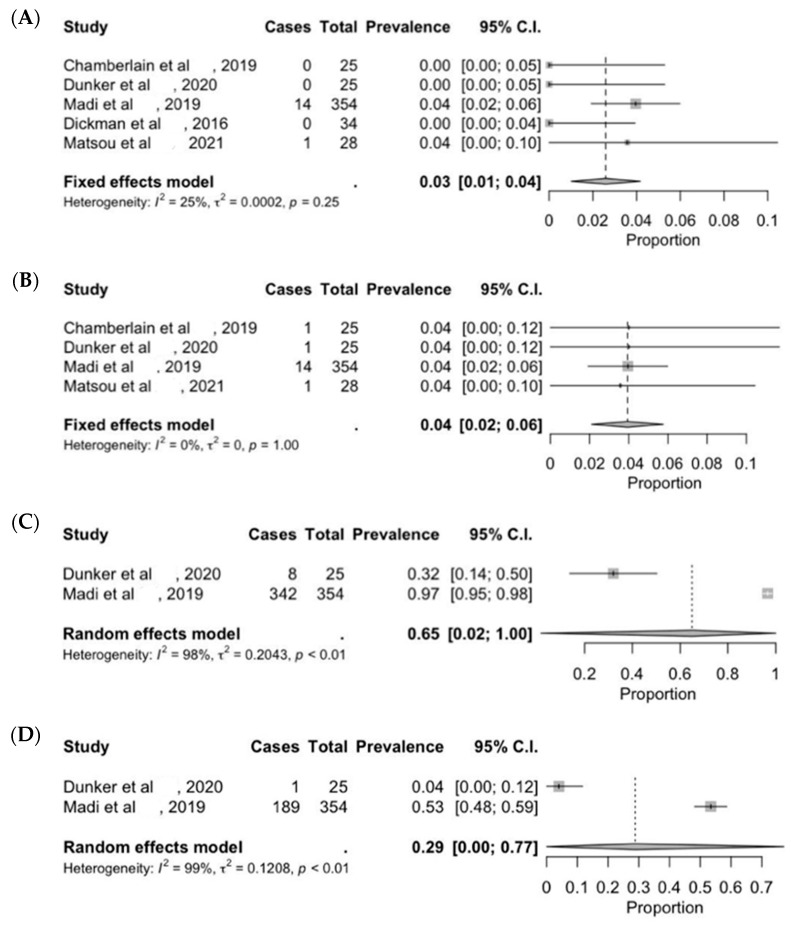
Forest plot diagram of UT-DSAEK clinical outcome: (**A**) rejection rate; (**B**) re-bubbling rate; (**C**) BSCVA > 20/30; (**D**) BSCVA 20/20.

**Figure 3:**
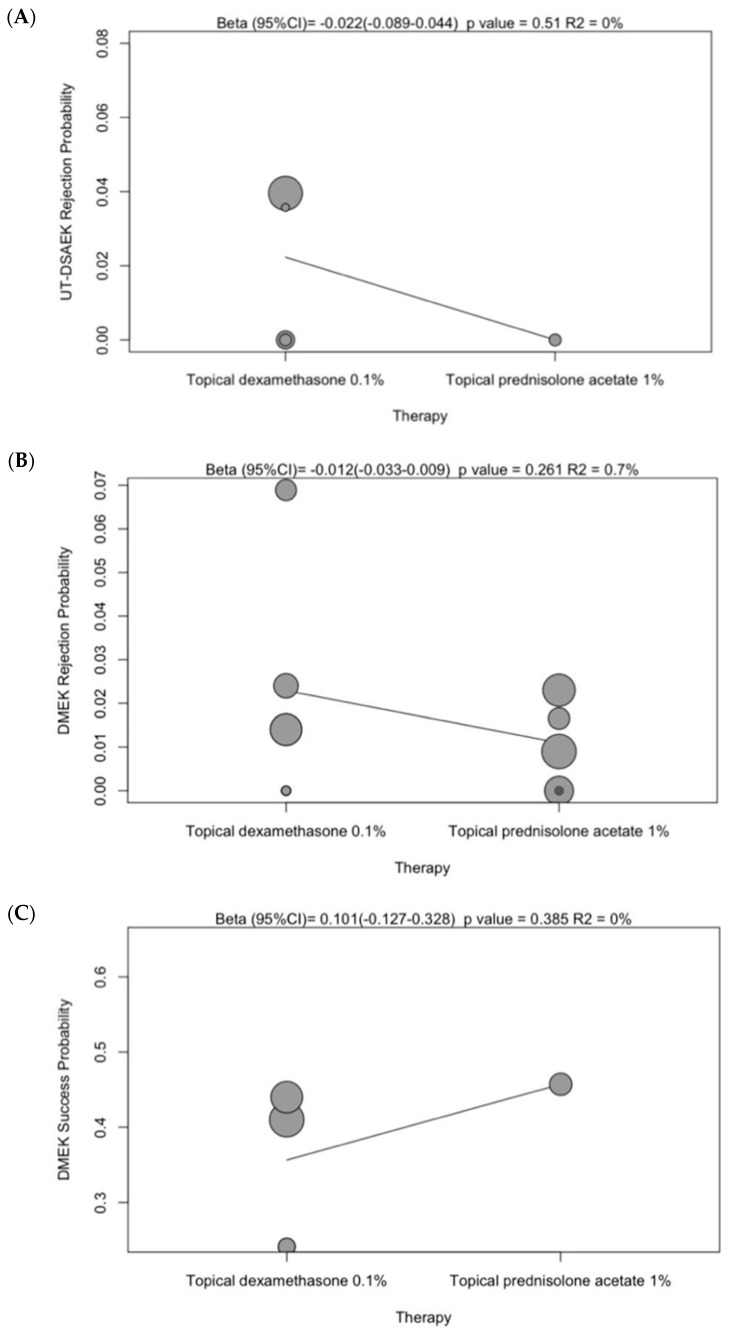
Metaregression diagram of dexamethasone and prednisolone effect: (**A**) UT-DSAEK rejection probability; (**B**) DMEK rejection probability; (**C**) DMEK success probability.

UT-DSAEK is the latest innovation in corneal transplant surgery, characterized by an ultra-thin corneal graft of 100 μm or less made by Descemet’s membrane, endothelium and a very small portion of stroma, obtained by microkeratome devices. Such innovation reduces the overall immunologic rejection to 3% (C.I. 1–4) (Figure 2A), although some studies report an increased risk to 6.9% at 5 years [39]. A proportion of 65% (C.I. 2–100) of patients reach a BSCVA of at least 20/30, while 29% (C.I. 0–77) achieve a BSCVA of 20/20 at the last follow up (Figure 2C,D) [37,38,51]. The procedure is the safest among the other EK alternatives with a low risk of graft detachment (4%; C.I. 2–6) (Figure 2B) [52]. Patients received one year of tapering postoperative corticosteroid eye drops, such as prednisolone acetate 1% or dexamethasone phosphate 0.1%, beginning from 4 times daily for 3 months [31,37,38]. No significant changes have been reported, but a reduction trend in rejection risk has been unveiled by our analysis (Figure 3).

**Figure 4:**
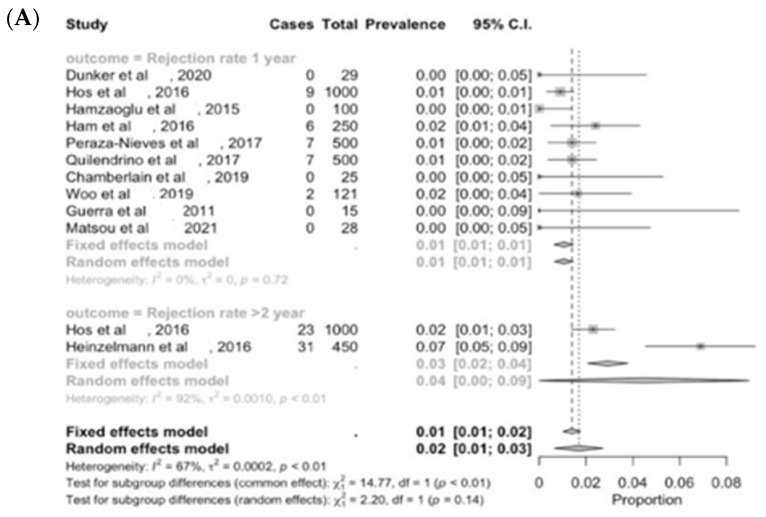

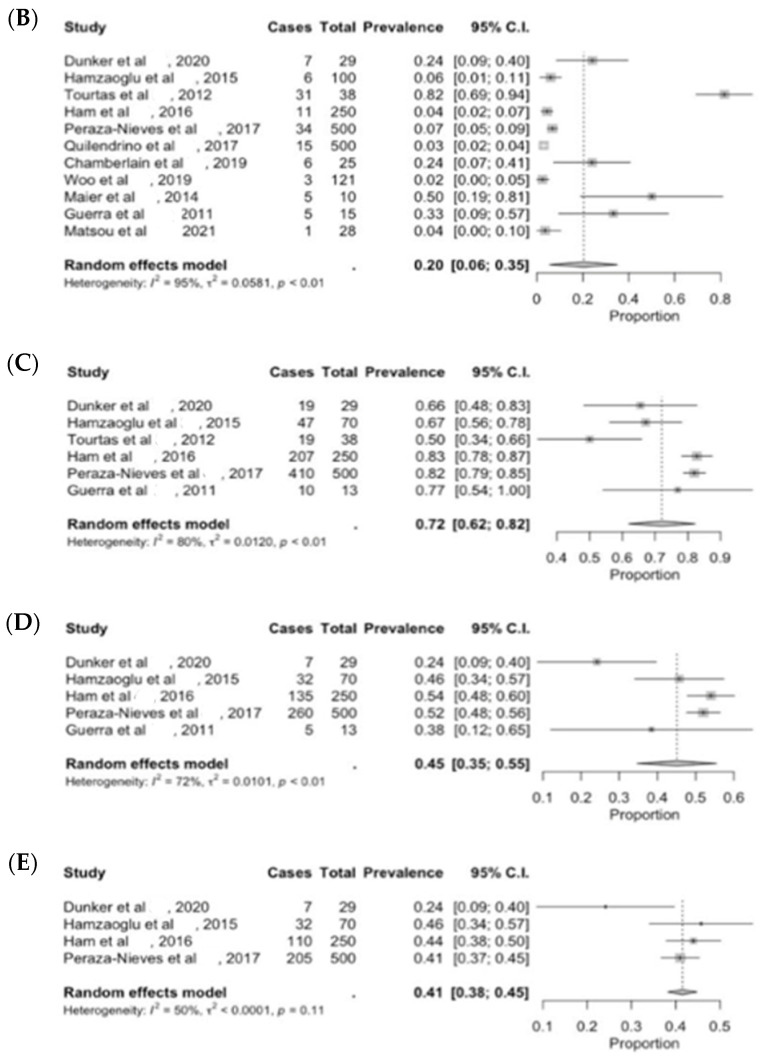
Forest plot diagram of DMEK clinical outcomes: (**A**) rejection rate; (**B**) re-bubbling rate; (**C**) BSCVA > 20/30; (**D**) BSCVA 20/20; (**E**) success rate.

DMEK is considered the most up to date EK procedure choice in Western countries [3], although the collection of inserted Descemet’s endothelium lamella (14–20 μm) requires a long learning curve [53,54]. Immune rejection is drastically cut down to 1% (C.I. 1–1) at 1 year and to 4% (C.I. 0–9) after more than two years from surgery (Figure 4A) [3,32,34,42]. Endothelial immune responses after DMEK may rarely have diffuse endothelial precipitates, Khodadoust line, anterior chamber reaction, and corneal edema; however, most patients with corneal graft rejection are asymptomatic, and several studies report that episodes can be underestimated [41].

A postoperative BSCVA of 20/20 is achieved by 41% (C.I. 38–45) of patients in 6 months, and a BSCVA of 20/20 is achieved by 45% (C.I. 35–55) at the last follow up (Figure 4D,E); 72% (C.I. 62–82) of total cases reached a BSCVA higher than 20/30 at the last follow up (Figure 4C) [42,43,55], though these cases experienced a greater need for re-bubbling, at around 20% (C.I. 6–35) (Figure 4B), compared to other EK procedures [33,52]. A mild post-DMEK hyperopic shift (around 0.6 diopters) has been reported, and it seems to be especially increasing in central flat or oblate posterior cornea [28,30,56,57,58].

A long-term graft rejection corticosteroid prophylaxis regimen is advised until at least the end of the second postoperative year [41,44]. In addition, a non-significant trend of better success rate and a reduced risk of rejection has been measured in DMEK patients when administering prednisolone acetate 1%. Dexamethasone phosphate 0.1% also shows to have a highly variable and less predictable effect in the different studies (Figure 3) [35,36,40].

In a direct comparative study sub-analysis, DMEK shows a better visual recovery, with odds ratios of 0.17 and 0.15 of patients achieving BSCVA 20/20 and 20/30, respectively. Mostly, DMEK shows a critically lower rejection risk (3.49 OR) compared to DSAEK. However, studies have measured a higher post-DMEK re-bubbling rate due to graft detachment (OR 0.11).

**Figure 5:**
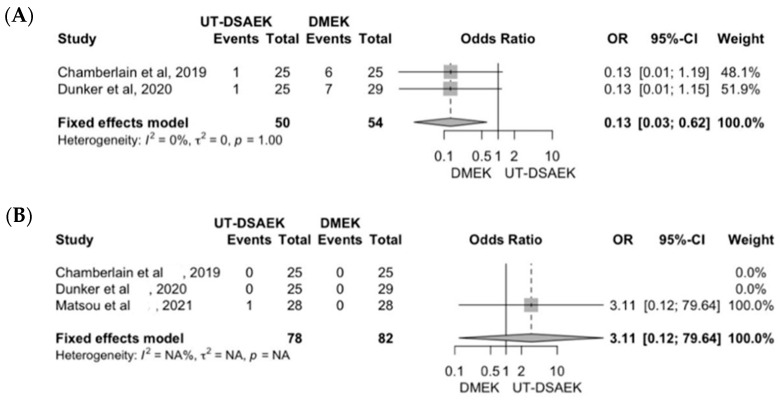
Comparative analysis of UT-DSAEK and DMEK clinical outcomes: (**A**) re-bubbling rate; (**B**) rejection rate.

A sub analysis directly comparing UT-DSAEK to DMEK demonstrated a lower risk of graft detachment (0.13 OR) in UT-DSAEK (Figure 5A) and a similar risk of immune rejection (Figure 5B). No visual recovery comparative studies have been carried out between two such techniques.

## 4. Discussion

Corneal transplantation represents a pinnacle in successful human tissue transplantation, with ongoing advancements aimed at enhancing graft survival [59,60]. Over time, techniques have evolved from penetrating keratoplasties (PK) to lamellar surgeries, driven by their multiple advantages, including a lower risk of rejection [15]. Within the cornea and anterior chamber, various active mechanisms of immune tolerance, including anterior chamber-associated immune deviation (ACAID), contribute significantly to transplantation success [61,62].

Despite these mechanisms, a notable percentage of cases still experience immune reactions leading to rejection and eventual graft failure [12,19]. While PK has historically been successful, it carries risks such as heightened rejection rates and anatomical complications. In contrast, endothelial keratoplasty (EK) offers a less invasive and safer alternative, preserving corneal integrity and lowering rejection rates. The widespread adoption of EK over PK, especially in cases of endothelial cell failure, underscores its efficacy [59]. However, in LR cases, surgical approaches are crucial for reducing the immunogenicity of transplanted tissue, thereby preventing immune reactions, rejection, and subsequent failure. EK techniques have evolved from Descemet’s stripping endothelial keratoplasties (DSEK) to Descemet’s membrane endothelial keratoplasty (DMEK), with the latter featuring regular, stroma-denuded grafts [14]. Nevertheless, debates persist regarding the performance, immunogenicity, and reproducibility of EK techniques, leading to a lack of clear guidelines for their alternative clinical applications [21,22].

Our research addresses this gap by exploring the clinical outcomes and rejection risks associated with post-operative prophylaxis in alternative EK procedures performed within corneal immunological sanctuaries.

Nowadays, DMEK lamellar surgery is the procedure of choice among alternative EK since it has a very fast visual recovery, such that almost half of patients have a 20/20 BSCVA within 6 months after the surgery. Moreover, two-thirds of the patients may reach a BSCVA higher than 20/30. Such a valuable visual outcome stands with a low risk of immune reaction and rejection in DMEK grafts, making the procedure simultaneously effective and safe. The immune reactions episodes are often mild and asymptomatic, even spontaneously self-resolving, and then not detectable. Thus, a higher immune reaction rate resulting from non-detectable episodes has been assumed [43]. The main limitation of DMEK surgery is related to the high rate of major complications. Post-operative graft detachment and dislocation, as well as intraoperative upside-down graft implantation, require further surgeries, such as re-bubbling, in almost one-fifth of all the cases.

UT-DSAEK aims to be an alternative endothelial transplantation procedure showing a similar rejection rate to DMEK but fewer intra- and post-operative complications, and a fast and flat learning curve.

DSAEK shows the highest rejection risk as well as worst visual outcomes among the procedures, and most of the surgeons would consider such a procedure in complicated cases, whereas DMEK is still considered too challenging.

EK procedures are less invasive procedures and reduce (1) the risk of ACAID disruption, (2) the amount of donor antigen presenting cells [60], and (3) the amount of immunogenic tissue. Hence, the risk of immune reaction after transplantation is much lower compared to penetrating keratoplasty (PK) [3,43,59,60,62]. In addition, the mini-invasive feature of surgical EK procedure, the limited suturing, and the mild postoperative inflammation may participate in the lower secondary immune reaction. However, a postoperative prophylactic regimen, particularly a long-term one of at least 2 years, of slowly tapering prednisolone acetate 1% eye drops seems to improve success, rejection, and subsequently survival rate in DMEK and UT-DSAEK but not in DSAEK.

Studies comparing DMEK with UT-DSAEK are scarce, and more long-term controlled randomized trials are required to confirm these results [54]. In fact, a critical selection bias may limit comparison among these procedures. In actual clinical practice, surgeons suggest DMEK mostly in eyes with normal ocular anatomy and good visual potential while they suggest UT-DSAEK in eyes with poor surgical view, complex ocular anatomy, and lower visual potential [53].

Finally, DMEK and UT-DSAEK have similar mid-term visual outcomes and rejection risk; UT-DSAEK shows a slow visual recovery compared to DMEK but a reduction in graft detachments and secondary interventions such as re-bubbling or regrafting.

## 5. Conclusions

In conclusion, this study demonstrates that DMEK has a faster visual recovery than UT-DSAEK but similar rejection rate and long-term visual acuity. Additionally, a one-year postoperative slow-tapering steroid regimen has a positive but not (yet) significant effect on rejection risk and visual outcomes.

## Figures and Tables

**Table 1:** Characteristics of the included studies.

Study	Year	Eyes	Design	Evidence Level
DSAEK				
Allan et al. [21]	2007	199	Retrospective case series	4
Li et al. [22]	2012	615	Retrospective case series	4
Price et al. [23]	2009	598	Retrospective study	4
Sepsakos et al. [24]	2016	400	Retrospective study	4
Li et al. [25]	2011	108	Retrospective case series	4
Wacker et al. [26]	2016	49	Prospective clinical trial	1c
Ezon et al. [27]	2013	122	Retrospective study	4
Hamzaoglu et al. [28]	2015	100	Retrospective case series	4
Wu et al. [29]	2012	353	Retrospective case series	4
Tourtas et al. [30]	2012	35	Retrospective case series	4
Dickman et al. [31]	2016	32	Randomized controlled clinical trial	1b
Woo et al. [32]	2019	423	Retrospective study	4
Maier et al. [33]	2015	10	Retrospective study	4
Heinzelmann et al. [34]	2016	89	Retrospective study	4
Guerra et al. [35]	2011	15	Retrospective case series	4
Gorovoy et al. [36]	2021	16	Retrospective study	4
UT-DSAEK				
Chamberlain et al. [37]	2019	25	Randomized controlled clinical trial	1b
Dunker et al. [38]	2020	25	Randomized controlled clinical trial	1b
Madi et al. [39]	2019	354	Retrospective case series	4
Dickman et al. [31]	2016	34	Randomized controlled clinical trial	1b
Matsou et al. [40]	2021	28	Randomized controlled clinical trial	1b
DMEK				
Dunker et al. [38]	2020	29	Randomized controlled clinical trial	1b
Hos et al. [41]	2017	1000	Retrospective case series	4
Hamzaoglu et al. [28]	2015	100	Retrospective case series	4
Tourtas et al. [30]	2012	38	Retrospective case series	4
Ham et al. [42]	2016	250	Retrospective case series	4
Peraza-Nieves et al. [43]	2017	500	Retrospective case series	4
Quilendrino et al. [44]	2017	500	Retrospective study	4
Chamberlain et al. [37]	2019	25	Randomized controlled clinical trial	1b
Woo et al. [32]	2019	121	Retrospective cohort study	2b
Maier et al. [33]	2015	10	Retrospective study	4
Heinzelmann et al. [34]	2016	450	Retrospective study	4
Guerra et al. [35]	2011	15	Retrospective case series	4
Matsou et al. [40]	2021	28	Randomized controlled clinical trial	1b

DSAEK: Descemet’s stripping automated endothelial keratoplasties; UT-DSAEK: ultra-thin Descemet’s stripping automated endothelial keratoplasty; DMEK: Descemet’s membrane endothelial keratoplasty.

**Table 2:** Patient’s clinical outcomes summary.

Treatment	Rejection Rate 1 Year		Rejection Rate > 2 Year		Re-Bubbling Rate		BSCVA > 20/30		BSCVA 20/20		Success Rate 6 Months	
	% (C.I)	n/eyes	% (C.I)	n/eyes	% (C.I)	n/eyes	% (C.I)	n/eyes	% (C.I)	n/eyes	% (C.I)	n/eyes
DSAEK	5 (2–7)	163/2735	13 (6–20)	200/1562	7 (0–15)	15/599	37 (14–60)	127/267	21 (2–39)	62/199	12 (7–17)	20/170
UT-DSAEK	3 (1–4)	15/466			4 (2–6)	17/432	65 (2–100)	350/379	29 (0–77)	190/379		
DMEK	1 (1–1)	31/2568	4 (0–9)	54/1450	20 (6–35)	124/1616	72 (62–82)	712/900	45 (35–55)	439/862	41 (38–45)	354/849

## Data Availability

Data are available upon request to the corresponding author, A.D.Z.

## Supplementary Materials

The following supporting information can be downloaded at: https://www.mdpi.com/article/10.3390/jcm13123431/s1, Figure S1: Prisma flow chart diagram.
