# Supplementary figures and images for: A Functional and Immunologic Point of View on Corneal Endothelial Transplantation: A Systematic Review and Meta-Analysis
Source: J Clin Med. 2024 Jun 12;13(12):3431. doi: 10.3390/jcm13123431 (PMC11204674; doi:10.3390/jcm13123431)

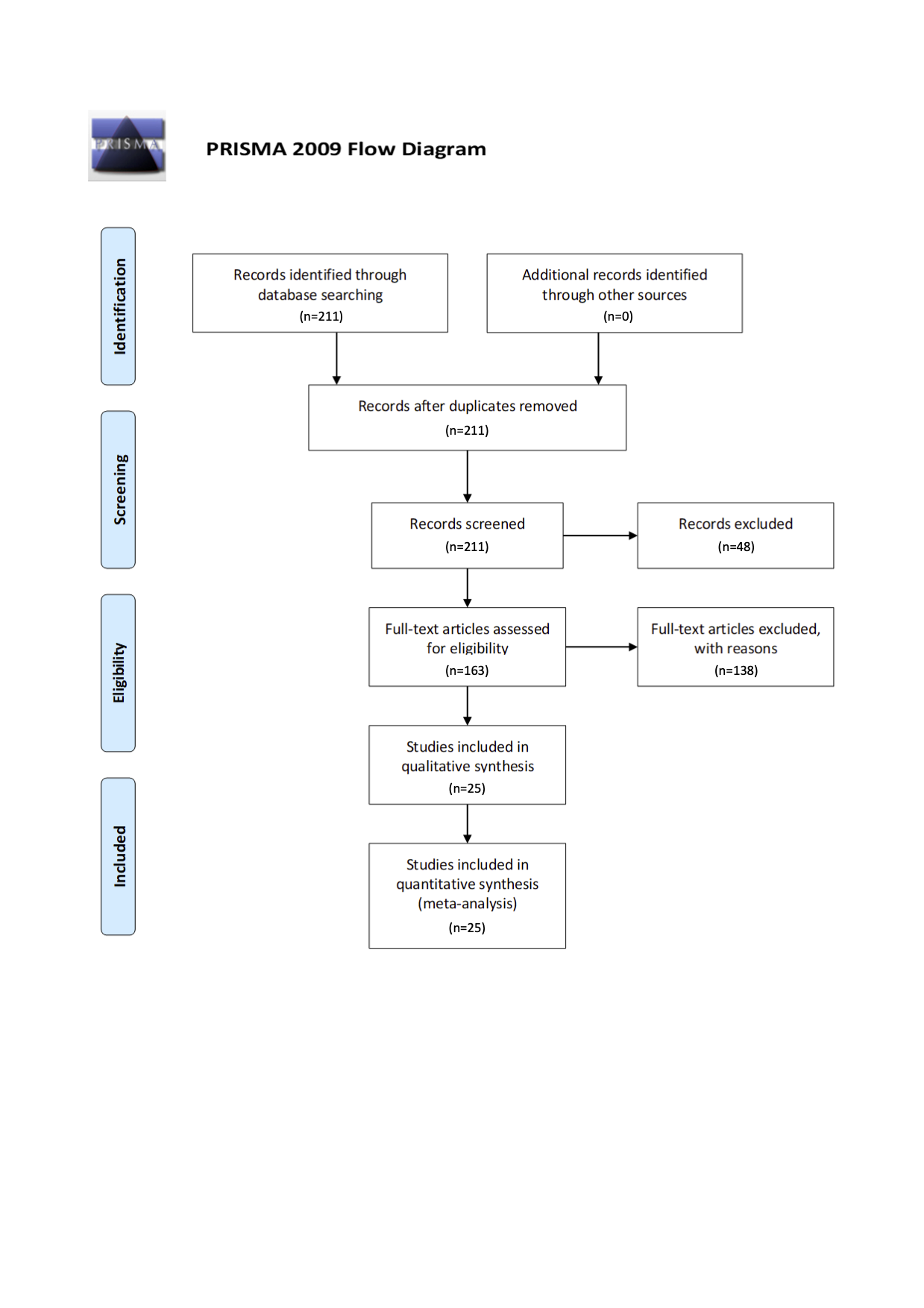

Supplement: Supplementary file 1 [file jcm-13-03431-s001.zip › Supplementary_Figure S1_Prisma_Diagram.tiff]
